# Thyroid Disease Prediction Using Selective Features and Machine Learning Techniques

**DOI:** 10.3390/cancers14163914

**Published:** 2022-08-13

**Authors:** Rajasekhar Chaganti, Furqan Rustam, Isabel De La Torre Díez, Juan Luis Vidal Mazón, Carmen Lili Rodríguez, Imran Ashraf

**Affiliations:** 1 Toyota Research Institute, Los Altos, CA 94022, USA; 2 Department of Software Engineering, School of System Sciences, University of Management and Technology, Lahore 54770, Pakistan; 3 Department of Signal Theory and Communications and Telematic Engineering, University of Valladolid, Paseo de Belén 15, 47011 Valladolid, Spain; 4 Higher Polytechnic School, Universidad Europea del Atlántico, Parque Científico y Tecnológico de Cantabria, Isabel Torres 21, 39011 Santander, Spain; 5 Project Department, Universidade Internacional do Cuanza, Cuito EN250, Bié, Angola; 6 Department of Project Management, Universidad Internacional Iberoamericana, Arecibo, PR 00613, USA; 7 Department of Project Management, Universidad Internacional Iberoamericana, Campeche 24560, Mexico; 8 Department of Information and Communication Engineering, Yeungnam University, Gyeongsan 38541, Korea

**Keywords:** machine learning, thyroid prediction, forward feature selection, bidirectional feature elimination

## Abstract

**Simple Summary:**

The study presents a thyroid disease prediction approach which utilizes random forest-based features to obtain high accuracy. The approach can obtain a 0.99 accuracy to predict ten thyroid diseases.

**Abstract:**

Thyroid disease prediction has emerged as an important task recently. Despite existing approaches for its diagnosis, often the target is binary classification, the used datasets are small-sized and results are not validated either. Predominantly, existing approaches focus on model optimization and the feature engineering part is less investigated. To overcome these limitations, this study presents an approach that investigates feature engineering for machine learning and deep learning models. Forward feature selection, backward feature elimination, bidirectional feature elimination, and machine learning-based feature selection using extra tree classifiers are adopted. The proposed approach can predict Hashimoto’s thyroiditis (primary hypothyroid), binding protein (increased binding protein), autoimmune thyroiditis (compensated hypothyroid), and non-thyroidal syndrome (NTIS) (concurrent non-thyroidal illness). Extensive experiments show that the extra tree classifier-based selected feature yields the best results with 0.99 accuracy and an F1 score when used with the random forest classifier. Results suggest that the machine learning models are a better choice for thyroid disease detection regarding the provided accuracy and the computational complexity. K-fold cross-validation and performance comparison with existing studies corroborate the superior performance of the proposed approach.

## 1. Introduction

Thyroid disease incidences have been on the rise in recent times. The thyroid gland has one of the most important functions in regulating metabolism. Irregularities in the thyroid gland can lead to different abnormalities; two of the most common are hyperthyroidism and hypothyroidism. A large number of people are diagnosed with thyroid diseases such as hypothyroidism and hyperthyroidism yearly [1]. The thyroid gland produces levothyroxine (T4) and triiodothyronine (T3) and insufficient thyroid hormones may lead to hypothyroidism and hyperthyroidism [2]. Many approaches are proposed to detect thyroid disease diagnosis in the literature. A proactive thyroid disease prediction is essential to properly treat the patient at the right time and save human lives and medical expenses. Due to the technological advancements in data processing and computation, machine learning and deep learning techniques are applied to predict the thyroid diagnosis in the early stages and classify the thyroid disease types hypothyroidism, hyperthyroidism, etc.

Due to the advancement in technologies such as data mining, big data, image and video processing, and parallel computing, the healthcare domain benefited from leveraging technology in many healthcare areas for human well-being [3]. The range of data mining-based health care applications may include the early detection of diseases and diagnosis, prediction of virus outbreaks, drug discovery and testing, health care data management, and patient personalized medicine recommendations, etc. [4]. Health care professionals strive to identify the diseases in the early stages so that proper treatment can be provided to the patients and cures the disease within a short time and with less expenditure. Thyroid disease is one of the diseases which impacts a sizeable human population worldwide. According to the world-leading professional association (American thyroid association), 20 million Americans have some form of thyroid disease [5]. Twelve percent of the US population is diagnosed with a thyroid condition at least once in a lifetime. These statistics signify that thyroid-based disease should not be taken lightly. Improving the health care practices to detect and prevent thyroid diseases using advanced technologies is highly desired.

Existing research works predominantly focus on binary classification problems where the subjects are classified into thyroid patients or health subjects, while multiclass-based detection works are only a few. Even for those, the focus is on three categories including normal, hypothyroidism, and hyperthyroidism. For the most part, the emphasis is placed on the optimization of machine learning and deep learning models and the feature selection part is under-studied or completely ignored for a thyroid disease problem. Despite the high accuracy reporting approaches, such approaches are tested on samples under 1000, and results are not validated. The classification in terms of the patient status like treatment condition, health condition, and general health issues based categorization is desired to predict the patient thyroid condition effectively and proactively treat the patient. Moreover, the performance comparison of machine learning and deep learning models is not carried out. This study aims at working on these issues and makes the following contributions

- A novel machine learning-based thyroid disease prediction approach is proposed that focus on the multi-class problem. Contrary to previous studies that focus on the binary or three-class problem, this study considers a five-class disease prediction problem.
- Four feature engineering approaches are investigated in this study to analyze their efficacy for the problem at hand. It includes forward feature selection (FFS), backward feature elimination (BFE), bidirectional feature elimination (BiDFE), and machine learning-based feature selection using an extra tree classifier.
- For experiments, five machine learning models are selected based on their reported performance for disease prediction, including random forest (RF), logistic regression, support vector machine (SVM), AdaBoost (ADA), and Gradient boosting machine (GBM). Moreover, three deep learning models are adopted as well, which include convolutional neural network, long short-term memory (LSTM) network, and CNN-LSTM. Performance is evaluated in terms of confusion matrix, 10-fold cross-validation, and standard deviation, in addition to accuracy, precision, recall, and F1 score.

The remainder of this article is organized as follows. Section 2 discusses the state-of-the-art works to detect and classify thyroid diseases. Section 3 presents the proposed methodology to address the thyroid disease prediction problem. This section also includes feature selection methods, machine learning techniques used in the article, and dataset description considered for this study. Section 4 describes the experimental results obtained in our study and comparison with prior art studies. Section 5 concludes the article with our contributions.

## 2. Literature Review

With recent technological advancements in data processing and computation, machine learning and deep learning techniques have been used in several research studies for thyroid disease prediction. Prediction of this disease at its early stages and its classification into cancer, Hypothyroidism, or Hyperthyroidism is helpful for timely treatment and recovery. The literature survey is performed using peer-reviewed article databases such as google scholar and Scopus. The searches were performed within the scope of the last five years to identify the recent works in our study. The keywords “Thyroid disease”, “Thyroid cancer”, “machine learning”, and “deep learning” combinations were used to select the relevant articles. As the number of retrieved results is much more for finding the relevant articles, we have further tuned the search queries and used a strict keyword search. Overall, more than 100 relevant articles were identified during our first screening. We further analyzed those articles and shortlisted 25 articles that are closely relevant to our work. Machine learning and deep learning methods are used both for thyroid disease detection and thyroid cancer detection. As the process of applying these methods is different for both tasks, they are discussed separately.

### 2.1. Thyroid Cancer Detection

The study [6] leveraged the least absolute shrinkage and selection operator (LASSO) and LR model to select the malignant thyroid nodule-associated ultrasonic characteristics. Then, RF is applied along with a scoring system to classify the malignant thyroid nodules. The logistic lasso regression (LLR) with RF obtained the best performance with 82% accuracy. Another study [7] performed machine learning-based prediction of the BRAF mutation presence in the confirmed cancer thyroid nodules. The authors selected 96 thyroid nodule ultrasonic images for this study. 86 radiomic features were extracted from the images, and three models, LR, SVM, and RF were applied to predict the presence of the BRAF mutation. The classification accuracy is reported as 64.3% for all three models. Idarraga et al. [8] performed machine learning-based thyroid nodule malignancy prediction using the ultrasonic and fine-needle aspiration (FNA) feature to avoid false-negative diagnosis in the early stages of thyroid cancer. The RF technique performed better than other techniques like decision tree (DT) and gradient descent (GD). All the above-mentioned works’ performance is not optimal to predict the thyroid cancer diagnosis and still has room for performance improvement.

### 2.2. Thyroid Disease Prediction

Several thyroid disease detection and classification approaches have been presented in the literature. For example, Garcia et al. [9] predicted the high probable molecules initiating the thyroid hormone homeostasis using machine learning algorithms RF, LR, GBM, SVM, and deep neural networks (DNN). The early prediction of the molecules is helpful for further testing in the first stages of thyroid disease. The molecular events were obtained from ToxCast datasets for running the experiments. The article reported that Thyroid Peroxidase (TPO) and Thyroid Hormone receptor (TR) achieved the best predictive performance with an F1 score of 0.83 and 0.81, respectively. The authors in [10] utilized the image processing techniques and feature selection methods to pick the important features from the dataset and achieve the best performance for thyroid disease prediction.

The thyroid disease classification is also a significant problem to be solved in the health industry. Razia et al. [11] compared the performance of various machine learning algorithms to classify Thyroid disease into normal, Hypothyroidism, or hyperthyroidism categories. The authors obtained the datasets from the University of California Irvine (UCI) machine learning library. The dataset contains 7200 samples, and each sample has 21 attributes. The authors reported that DT outperformed the SVM, NB, and multilinear regression (MLR) with 99.23%. However, multi-classification is limited to three categories, and limited information is provided on data preprocessing to assess the applicability of the results for real-time datasets. A multi-kernel SVM is proposed in the paper [12] to classify thyroid diseases. The authors mentioned that the multi-kernel SVM achieved 97.49% performance accuracy on UCI thyroid datasets. The improved gray wolf optimization performs the feature selection and enhances the performance.

A study [13] performed multiclass hypothyroidism using selective features and machine learning algorithms. Hypothyroidism is classified into four categories. The results show that RF performed well with 99.81% accuracy compared to the SVM, KNN, and DT algorithms. However, the authors did not mention the performance of their proposed methodology for thyroid disease classification. Another study [14] tested three feature selection methods along with SVM, DT, RF, LR, and Naive Bayes (NB) to make early predictions for hypothyroidism. Three feature selection methods, recursive feature selection (RFE), univariate feature selection (UFS), and principal component analysis (PCA), are tested in combination with ML algorithms. The RFE combination with ML algorithms performed better than other feature selection methods. All the five ML algorithms obtained 99.35% accuracy when combined with RFE feature selection. However, the data sample size is very small, with only 519 records. A large-scale dataset is needed to evaluate the effectiveness of their method.

The authors [15] evaluated the performance of the thyroid disease classification using various machine learning algorithms. SVM, RF, DT, NB, LR, K nearest neighbor (KNN), and MLP are used for disease prediction. A dataset sample of 1250 is taken from hospitals and laboratories in Iraq. The MLP predicted the thyroid classification with 96.4% accuracy. However, there is still room for performance improvement. Hosseinzadeh et al. [16] proposed a multiple multi-layer perception (MMLP) technique to classify thyroid diseases. When the MMLP is applied along with a set of six networks, the accuracy is improved by 0.7% compared to a single MLP. Although MMLP obtained 99% classification accuracy on large dataset samples, training deep learning techniques like MMLP is costly and needs high computational resources to train faster. The KNN with various distance functions is implemented to test the thyroid disease detection in [17]. The chi-square and L1-based featured selection methods were used to select the optimal features before applying the KNN with Euclidean and Cosine distances. The authors reported that KNN obtained promising results. However, the tested sample size is very small, with 590 samples in total.

Mishra et al. [18] applied the ML techniques sequential minimal optimization (SMO), DT, RF, and K-star classifier to predict hypothyroid disease. A sample size of unique 3772 records is considered for this study. The authors reported that RF and DT performed better than the other two techniques, with accuracy scores of 99.44% and 98.97%. However, the authors did not consider hyperthyroid predication. Alyas et al. [19] performed a comparative analysis of the machine learning techniques DT, RF, KNN, and artificial neural network (ANN) to detect thyroid disease. The tests were conducted on the largest dataset and considered both sampled and unsampled data for thyroid disease prediction. RF obtained the best prediction with 94.8% accuracy. However, the authors did not perform the thyroid disease type prediction tests. Researchers also applied deep learning models to predict thyroid disease classification. For instance, the authors [20] used a deep neural network (DNN) to predict the thyroid disease classification. The performance evaluation is done on the UCI dataset of 3152 unique samples. The authors reported 99.95% accuracy when using DNN to classify thyroid disease. However, a large dataset is required to train the model for performance evaluation properly. Additionally, more computing resources are needed to train the deep learning models.

Table 1 provides the comparative analysis of the existing works discussed in this section. Various datasets are used in the literature to evaluate the performance of thyroid disease detection. However, most of the datasets given in Table 1 are not standard datasets for performance evaluation and comparison with the existing work. Therefore, we elected a well-known UCI dataset for our study. Although tremendous work has been done in the above studies with high accuracy results to detect and classify thyroid disease, detailed research on the feature selection is not well explored for thyroid disease classification problems. Besides, the performance results reported in the context of thyroid disease classification accuracy are insufficient, and there is still scope for improvement. Furthermore, all the prior works classify thyroid problems into three categories (normal, hypothyroidism, or hyperthyroidism). The classification in terms of the patient status like treatment condition, health condition, and general health issues based categorization is desired to predict the patient thyroid condition effectively and proactively to treat the patient. Moreover, the detailed evaluation of the machine learning and deep learning-based techniques for thyroid disease classification and their performance comparison is not well discussed in the state-of-the-art. So, we propose a feature selection-based, highly accurate, multiclass supportive thyroid disease classification solution to overcome those limitations and provide a detailed performance comparison of machine learning and deep learning-based solutions.

## 3. Proposed Methodology

**Figure 1:**
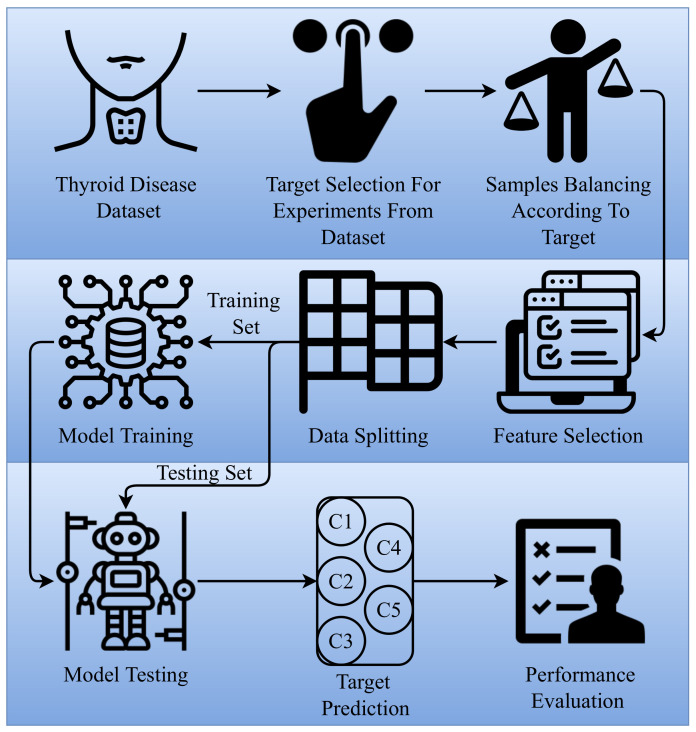
Flow of the proposed methodology.

Figure 1 shows the architecture and flow of the proposed approach for thyroid disease prediction. First, we acquired the disease dataset from UCI (a famous data repository). The dataset consists of several thyroid-related disease records and many target classes. The samples for target classes are few, which are not enough to train models, so we select only those target classes whose samples are more than 250, as a result, we got five target classes. After selecting the target classes for experiments, we performed the data balancing. Normal class samples were 6771 in total, which is more compared to other target class samples, so we randomly selected only 400 samples for the normal class to make dataset balance. It is followed by the feature selection process, where many feature selection techniques are applied. Experiments are performed with an 80–20 train–test split using several machine learning and deep learning models.

### 3.1. Dataset Acquisition

The datasets created for our study are obtained from the UCI thyroid disease datasets. The UCI machine learning repository maintains a variety of thyroid disease datasets [22]. The dataset contains 9172 sample observations and each sample is represented by 31 features. Table 2 presents the dataset description of the UCI thyroid dataset.

The target classification contains health conditions and diagnosis classes. The importance of the features should be estimated to elect the optimum number of features for thyroid disease classification. As we can see in Table 3, the 31 features include Boolean, float, int, and string types. The feature-based analysis is performed to estimate the importance of the features.

Table 4 shows the dataset thyroid health condition state and the diagnosis class. The class counts clearly show that the dataset is highly imbalanced. For instance, most of the samples in the dataset do not belong to any particular class. Therefore, the data preprocessing is performed to obtain the standard dataset for our performance evaluation. As described in the proposed methodology subsection, the feature selection and feature preprocessing yield the balanced thyroid disease classification dataset. The majority of the classification count is categorized as “no condition”. The “no condition” means that the data sample is not categorized as any other classes like hyperthyroid, hypothyroid, binding proteins, general health, replacement therapy, antithyroid treatment, or miscellaneous. The patients classified as “no condition” means normal patients who do not have thyroid disease. On the other hand, concurrent non-thyroidal illness is commonly seen in critically ill patients with chronic illness, and the serum thyroid levels change due to the chronic illness. The non-thyroidal illness may occur in the absence of hypothalamic-pituitary-thyroid primary dysfunction [23].

The dataset consists of 9173 patient records. The 6771 records are normal patient records and do not show any sign of thyroid disease. The other notable patient condition records include 233 primary hypothyroid, 359 compensated hypothyroid patients, 346 patients with increasing binding proteins, and 456 concurrent non-thyroidal illness patients.

Table 5 displays the dataset target classification categories and the sample counts for each category. A 400 sample count was randomly picked from a pool of 6771 normal category sample records to balance the dataset. The other categories such as increased binding protein, primary hypothyroid, compensated hypothyroid, and concurrent non-thyroidal illness counts remain unchanged. Since the number of samples for each class is not the same, we performed the dataset balancing by randomly selecting 400 samples for the normal class while other classes with at least 200 samples are selected. The balanced dataset is shown in Table 5 and samples of the dataset are shown in Table 6.

A blood test is one of the ways to diagnose hypothyroidism, but after a lab blood test, a medical expert needs to examine the test stats of hormones and other parameters of the patient to diagnose the disease. There is very little difference in the blood test stats, which refer to different thyroid hormone levels. Table 6 shows the data for three target classes and we can see there is a very small difference in some features for two different target classes. Such minor differences can lead to the wrong diagnosis even by medical experts as human error is expected. Incorrect diagnosis may lead to wrong medication and further complexities. So, an automated system can be very helpful to assist medical experts and even make automated disease predictions without any human mistakes. So, this study follows a machine learning approach to make automatic predictions for different thyroid diseases.

### 3.2. Feature Selection

**Figure 2:**
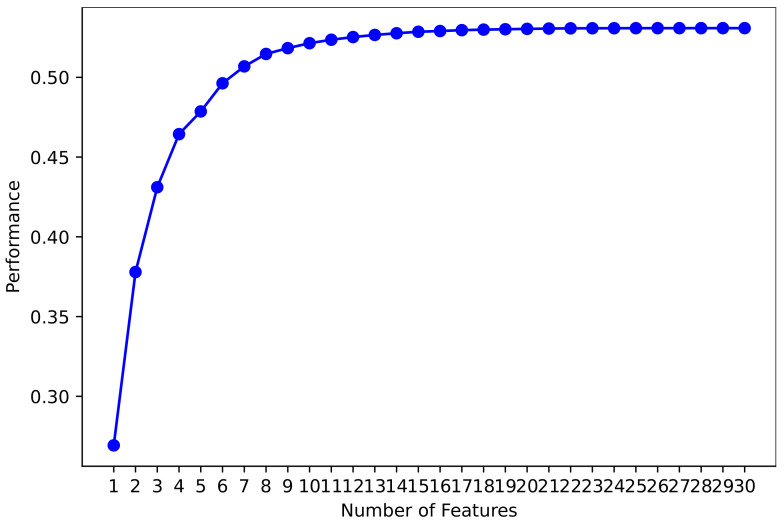
Feature impact on models performance.

The dataset consists of 30 features and some features are not important for the good-fit of learning models to improve the performance of machine learning models, as shown in Figure 2. We deployed several feature selection techniques such as forward feature selection, backward feature elimination, bi-directional elimination, and machine learning feature selection. These techniques help to extract the important features from the dataset to train the machine learning models.

In machine learning, feature selection is crucial to designing a good model and obtaining the best model performances [24]. The redundant and undesired features may need to be removed from the original datasets to train the model faster, easily interpret the data, and avoid overfitting problems. We have considered the wrapper method for feature selection, as determining the right set of features for thyroid disease classification is essential. The feature selection is based on the specific ML algorithm used to fit the dataset in the wrapper method. A greedy selection method selects the combination of feature sets and evaluates the performance of the feature set combinations against the evaluation criteria. The evaluation criteria may include metrics such as *p*-value, accuracy, F1-score, etc., to assess the performance of feature set combinations. The detailed description of the selected four feature selection techniques and machine learning feature selection is as follows.

#### 3.2.1. Forward Feature Selection

In FFS [25], we start with a null model and then try to fit the model with each feature value. The feature with a low *p*-value is selected for the next round. Then, we start fitting the model with two feature combinations. The minimum *p*-value feature set in the first round should be the one feature candidate when fitting the models with two feature combinations. The low *p*-value of two features is considered for fitting the model with three feature combinations. This process is repeated until the minimum *p*-value for each feature in the feature set is less than the significance level.

Step 1: Choose the significance level value (S) and start with null set [26].
  (1) $$Y_{0}=\left\{\phi \right\}$$
Step 2: Select the first feature using some criteria. For example, pick a random feature from the list of features. The below equation represents the selection of minimum *p*-value feature selection out of all the features used for selection.
  (2) $$X^{+}=\underset{x\notin Y_{k}}{arg max}J\left(Y_{k}+x\right)$$
Step 3: The identified minimum *p*-value feature is updated to the list of all the existing minimum *p*-value features. The iteration *k* value is incremented by 1. At this point, repeat, go back to step 2, and continue the process until all the feature’s *p*-value is less than the Significance level. The iterative process stops when $Y_{k}<S$ and the *k* value is the total number of features.
  (3) $$Y_{k}=Y_{k}+X^{+};k=k+1$$

This study deploys the FFS due to its wide adaptation in the existing literature. It is applied by passing the original dataset. FFS is deployed with a significance level of 0.05 with 95% confidence.

#### 3.2.2. Backward Feature Elimination

In BFE, we start with a model with all the features. The highest *p*-value feature is selected to be removed from the model and then fit the model. The removed feature *p*-value must be greater than the significance level value. This process is repeated until all the high *p*-value features are eliminated from the model while ensuring that all the eliminated features *p*-value greater than the significance level. By the end of this process, the final set of the existed features are the most relevant and valuable features used for accurate detection and classification.

Step 1: Start with all features to fit the model.
  (4) $$Y_{0}=X$$
Step 2: Identify the high *p*-value feature from the feature list. The high *p*-value feature is compared with the significance level value (*S*). The condition x>S should be satisfied to consider the feature for elimination [26].
  (5) $$X^{-}=\underset{x\in Y_{k}}{arg max}J\left(Y_{k}-x\right)$$
Step 3: The high *p*-value is removed from the list and goes back to step 2 to perform the next iteration (k+1) feature elimination. When the *k* value is zero, the final list of features represents the selected feature list using BFE.
  (6) $$Y_{k-1}=Y_{k}-X^{-};k=k+1$$

BFE is another widely used feature selection approach in the literature. BFE technique is deployed with a significance level of 0.05 with a 95% confidence.

#### 3.2.3. Bi-Directional Elimination

The BiDFE method combines the forward feature selection and backward feature elimination methods. This method is similar to forward feature selection. But, when the new feature is selected, the backward elimination process kicks it to compare it with previously selected features. Suppose any previously chosen features with a *p*-value is greater than the defined significance level ‘out’ value is eliminated. In this method, two significance level values should be determined with ‘in’ and ‘out’ of value ranges. The feature *p*-value should be less than the significance level inner value to include in the feature selection and greater than the significance level outer value to exclude the feature from the feature list.

Step 1: We start with an empty set. Initially, a feature is selected based on the defined criteria. We use the forward feature selection to include the features in the list [26].
  (7) $$Y_{F}=\left\{\phi \right\};Y_{B}=X$$
Step 2: The next best feature is selected using the *p*-value comparison. A typical forward feature selection process is followed to select the essential features.
  (8) $$X^{+}=\underset{\begin{array}{c} x\notin Y_{F_{k}}\\ x\in Y_{B_{k}} \end{array}}{arg max}J\left(Y_{F_{k}}+x\right);$$
  (9) $$Y_{F_{k}+1}=Y_{F_{k}}+X^{+};$$
Step 3: The next best feature is selected using the *p*-value comparison. A typical forward feature selection process is followed to choose the next feature; then backward feature elimination process kicks in to eliminate any selected features that are unimportant. We can go back to step 2 to repeat this process and continue until the k value reaches the total number of features count.
  (10) $$X^{-}=\underset{\begin{array}{c} x\in Y_{B_{k}}\\ x\notin Y_{F_{k+1}} \end{array}}{arg max}J\left(Y_{B_{k}}-x\right);$$
  (11) $$Y_{B_{k}+1}=Y_{B_{k}}-X^{-};k=k+1$$

BiDFE is deployed with significance level in = 0.05, significance level out = 0.05, and 95% confidence.

#### 3.2.4. Machine Learning Feature Selection

Machine learning-based methods, especially ensemble techniques, are used to select the essential features. We have considered the extra tree classifier technique as one of the feature selection methods in this work [27,28]. The extra tree classifier randomly constructs multiple decision trees using the training dataset. The splitting of the nodes in the decision tree is followed by either the Gini index or entropy criteria. The Equation (12) is used to measure the entropy. The value *c* indicates the unique class labels, and the $p_{i}$ is the fraction of the rows containing the label *i* in the dataset.

(12) $$Entropy\left(E\right)=-\Sigma_{i=1}^{C}p_{i}\log_{2}\left(p_{i}\right)$$

**Figure 3:**
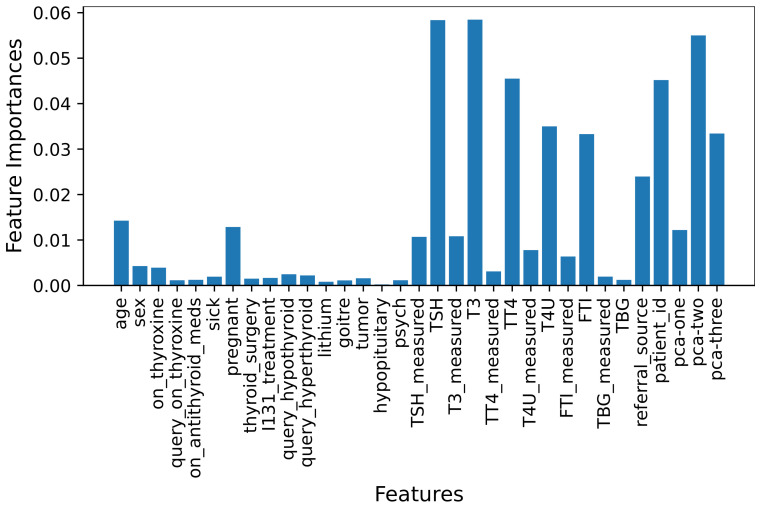
Feature Importance using MLFS.

Entropy measures the information about the disorder of the features with the target. We have considered the entropy criteria in our feature selection process. The entropy of obtained features from each decision is determined, and the cumulative entropy values for each feature are used to find the important features. The set of high entropy features is considered to be the shortlisted features. Figure 3 shows the features’ importance using MLFS.

For MLFS, we used an ETC classifier with n_estimators = 200, max_depth = 20, which found the importance of each feature and ranked them, then we selected the score > 0.015 importance features for learning models training.

### 3.3. Machine Learning Models

This study employs several machine learning models for thyroid disease detection. RF, LR, SVM, ADA, and GBM are applied to the problem at hand. These models are fine-tuned to optimize their performance. For that, several hyperparameters are optimized. Details of hyperparameter settings of the models are given in Table 7.

## 4. Results and Discussion

This section presents the details of experiments on thyroid disease prediction using machine learning. We discuss the results with each feature selection technique using machine learning and deep learning models. We split the dataset into training and testing sets with an 80:20 ratio, where we used 80% of the data for model training and 20% of the data for model testing. The ratio of the target with respect to each target class is shown in Table 8.

After data splitting, we used several machine learning and deep learning models with their best hyperparameter settings. Models are trained with important features selected by feature selection techniques and then evaluated using 20% test data and 10-fold cross-validation techniques. We evaluate models in terms of accuracy, precision, recall, F1 score, confusion matrix, and standard deviation (SD).

### 4.1. Results Using Original Feature Set

Table 9 shows the results of machine learning models using the original feature set. Models perform well in terms of all evaluation parameters such as tree-based models RF, GBM and ADA are good with 0.98, 0.97, and 0.97 accuracy scores, respectively. The tree-based ensemble can perform better even on the small feature set and small size of the dataset. While linear models such as LR and SVM are poor in performance because of the small size of the feature set and dataset. LR and SVM both show similar performance, each with a 0.85 accuracy score. Overall, RF is good with the original dataset in terms of accuracy score in comparison with all other used models.

### 4.2. Performance of Models with FFS

**Figure 4:**
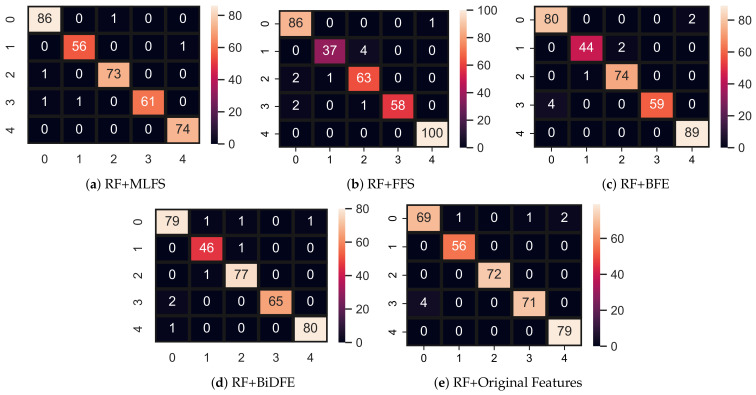
Feature space using different feature selection methods.

Models’ performance using FFS is shown in Table 10 and according to the results, only SVM improves its performance from 0.85 to 0.92 because, with the selected feature, data become more linearly separable, which helps SVM to draw hyperplane with a good margin to classify the data. The tree-based model ADA drops its accuracy from 0.97 to 0.93 and LR drops from 0.85 to 0.83 accuracy because they require a large feature set for a good fit. Overall, FFS does not help to improve models’ performance, so we try models with other feature selection approaches. Figure 4a–e show the confusion matrix with all approaches. RF gives a total of 344 correct predictions out of 355 predictions and 11 wrong predictions using FFS as shown in Figure 4b.

### 4.3. Results Using BFE Features

**Figure 5:**
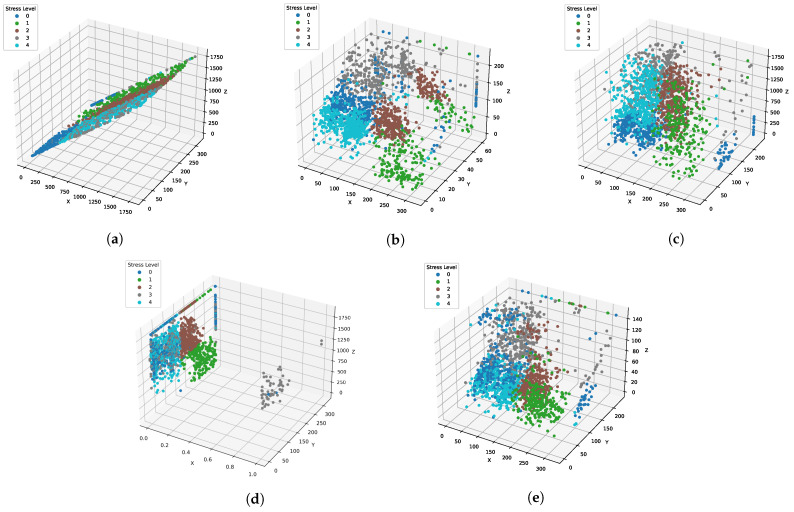
Feature Space using Different Feature Selection Methods. (**a**) ML. (**b**) Forward Feature Selection (FFS). (**c**) Backward Feature Elimination (BFE). (**d**) Bi-Directional Feature Elimination (BiDFE). (**e**) Original.

Table 11 shows the results of machine learning models using the BFE technique. Results indicate that reducing the feature set size also reduces the performance of learning models. All models drop their accuracy score and other evaluation scores with BFE techniques which shows that the selected features by BFE are not suitable for models good-fit because, with this feature set, data are not linearly separable, as shown in Figure 5. RF gives a total of 346 correct predictions out of 355 predictions and 9 wrong predictions using BFE, as shown in Figure 4c.

### 4.4. Models’ Performance Using BiDFE Features

Table 12 shows the performance of machine learning models using BiDFE. All models are good with BiDFE in comparison with FFS and BFE feature selection techniques. RF achieved a significantly better accuracy of 0.98 and GBM is just behind the RF with a 0.96 accuracy. LR and ADA are poor in accuracy scores which shows that it is not effective for those models as they require a large feature set. RF gives a total of 347 correct predictions out of 355 predictions and 8 wrong predictions using BiDFE, as shown in Figure 4c.

### 4.5. Performance of Models Using MLFS Features

Models’ performance using the ML feature selection technique is shown in Table 13. Models are significant with this approach as RF achieved the highest accuracy of this study 0.99 with MLFS. Other models such as GBM also achieved their highest accuracy of 0.98 and LR has a 0.87 accuracy score which shows the significance of MLFS. The models’ performance is significant with MLFS because this technique selects the feature based on how much a feature is correlated to its target. High correlation means more important features. This significance of MLFS selects a small but efficient feature set to train machine learning models. RF gives a total of 350 correct predictions out of 355 predictions and five wrong predictions using the MLFS technique, as shown in Figure 4e.

### 4.6. K-Fold Cross-Validation for Models

We also evaluate all models in terms of 10-fold cross-validation to show the significance of the proposed approach for thyroid disease prediction. Table 14 shows the results of models with all feature selection techniques. Models with MLFS outperformed those with 10-fold cross-validation, such as RF achieved a significant 0.94 accuracy with 0.01 SD and SVM achieved 0.91 accuracy with 0.13 SD. This shows the significance of MLFS as compared to other feature selection techniques. Table 14 also shows the computational cost of machine learning models in terms of time (seconds). In a significant approach, RF+MLFS computational time is only 1.689 s. LR has the lowest computational cost, but its low accuracy score makes it inefficient to be used for thyroid disease prediction. SVM is a much more expensive choice in terms of computational cost and its accuracy is also low as compared to tree-based models. RF is best in terms of computational time and accuracy score, which make it significant for the proposed approach.

### 4.7. Deep Learning Models Results

The performance of deep learning models is also evaluated on the used dataset with each feature selection technique. We used several deep learning models in comparison with machine learning models such as LSTM, CNN, and CNN-LSTM. These models are used with state-of-the-art architectures, as shown in Table 15.

**Figure 6:**
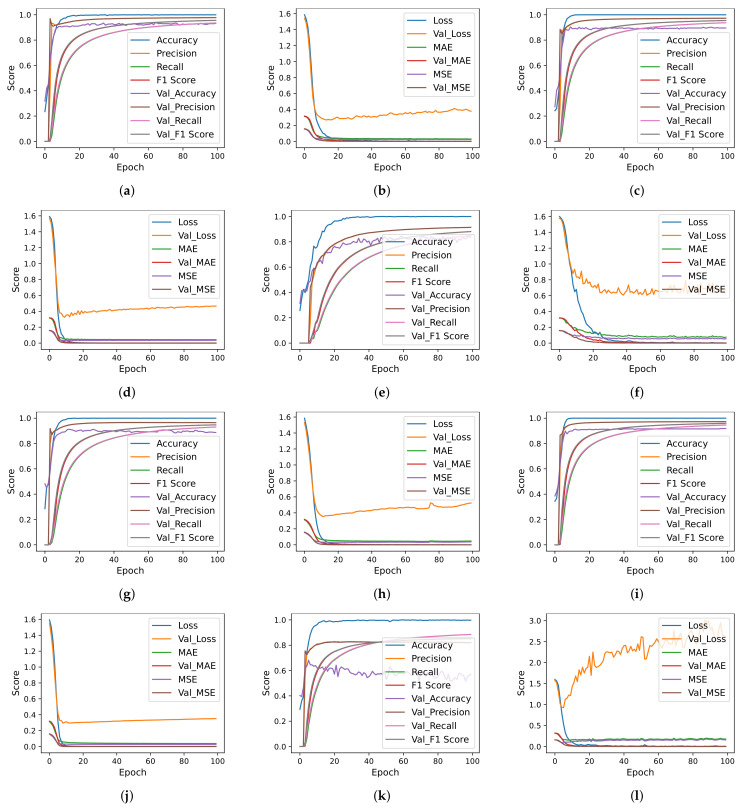
Deep learning models per epochs evaluation scores using original features and MLFS. (**a**) CNN Accuracy using Original Features, (**b**) CNN Loss using Original Features, (**c**) CNN-LSTM Accuracy using Original Features, (**d**) CNN-LSTM Loss using Original Features, (**e**) LSTM Accuracy using Original Features, (**f**) CNN Loss using Original Features, (**g**) CNN Accuracy using MLFS, (**h**) CNN Loss using MLFS, (**i**) CNN-LSTM Accuracy using MLFS, (**j**) CNN-LSTM Loss using MLFS, (**k**) LSTM Accuracy using MLFS, and (**l**) LSTM Loss using MLFS.

**Figure 7:**
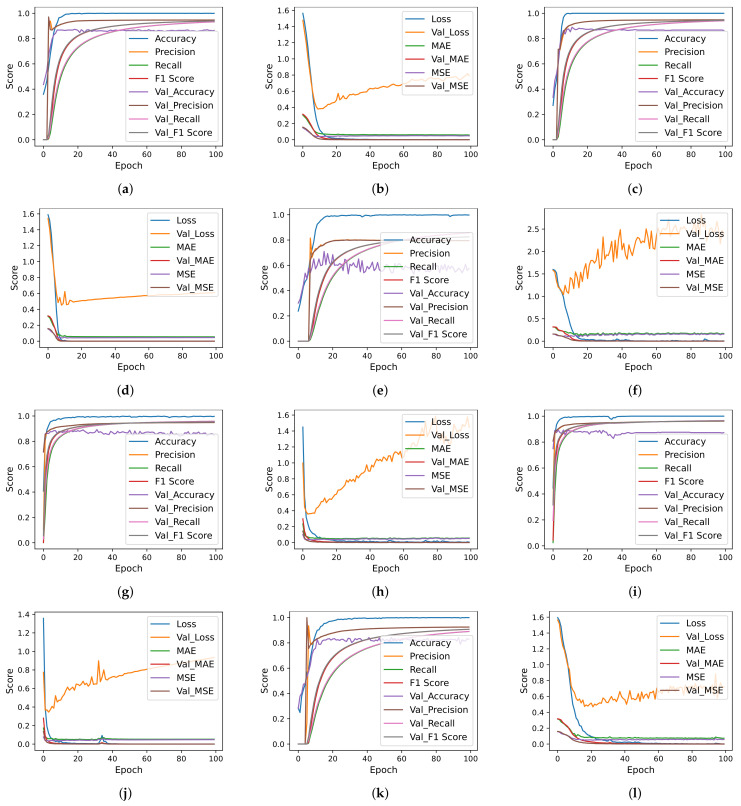
Deep learning models per epochs evaluation scores using BiDFE and BFE. (**a**) CNN accuracy using BFE, (**b**) CNN loss using BFE, (**c**) CNN-LSTM accuracy using BFE, (**d**) CNN-LSTM loss using BFE, (**e**) LSTM accuracy using BFE, (**f**) LSTM loss using BFE, (**g**) CNN accuracy using BiDFE, (**h**) CNN loss using BiDFE, (**i**) CNN-LSTM accuracy using BiDFE, (**j**) CNN-LSTM loss using BiDFE, (**k**) LSTM accuracy using BiDFE and (**l**) LSTM loss using BiDFE.

**Figure 8:**
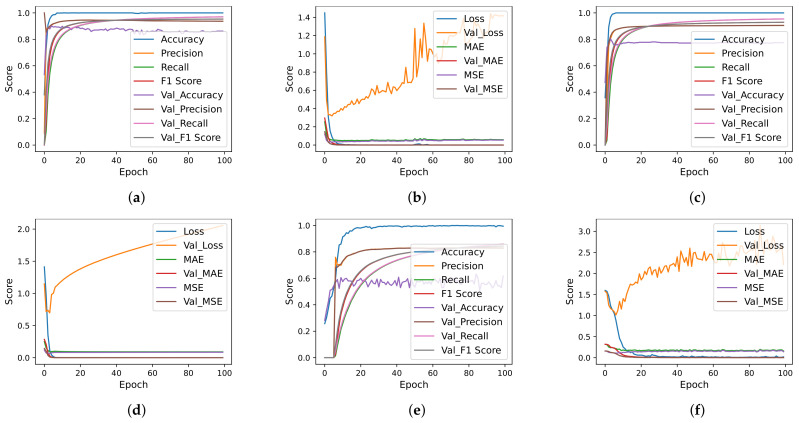
Deep learning models per epochs evaluation scores using FFS. (**a**) CNN accuracy using FFS, (**b**) CNN loss using FFS, (**c**) CNN-LSTM accuracy using FFS, (**d**) CNN-LSTM loss using FFS, (**e**) LSTM accuracy using FFS and (**f**) LSTM loss using FFS.

Deep learning models are designed with different numbers of layers, dropout layer position, number of neurons, and activation functions. Each model is trained using the ‘categorical_crossentropy’ loss function, while the ‘Adam’ optimizer is used. The models are trained with a 16 bath and 100 epochs are used for training. Figure 6, Figure 7 and Figure 8 show the per epochs evaluation score for each model using each feature selection technique.

Overall the performance of deep learning models is not as good as machine learning models because of the small feature set size. Deep learning models require a large feature set for a good fit. Table 16 shows the results of all deep learning models and significant results with both the original feature set and MLFS. The original set is large, which is the reason CNN achieved a 0.93 accuracy score while MLFS is significant and CNN-LSTM achieved 0.92 accuracy with these features. Machine learning models are good in performance because they do not require a large feature set, while deep learning requires a large feature set.

Table 17 shows the computational cost of deep learning models. Computational time taken by deep learning models is more as compared to machine learning models, while accuracy is low of deep learning models as compared to machine learning models. Overall, all results and analysis show that machine learning models are better in terms of both accuracy and efficiency. This is because of the small feature set and small dataset.

### 4.8. Limitations of Current Study

We analyze that the feature selection technique can be effective in improving the results, but it also reduces the size of data which is not good for linear models. This small feature set after the selection of important features is a limitation of this study. Another limitation is the small size of data which is not enough to train deep learning models. We worked on a few target classes because of fewer samples available for other target classes, which is also a limitation of this study; however, existing literature often considers only three classes, while this study uses five target classes. We will consider all these limitations in our future work to improve thyroid disease prediction accuracy and efficiency.

### 4.9. Comparison with Other Studies

To show the significance of our proposed approach, we have made a comparison with existing studies. We select recent studies that worked on disease prediction using categorical or numerical datasets. We deployed the proposed approaches on our used dataset and evaluated the previous studies’ models in terms of accuracy and F1 score. We did not deploy the feature selection approach with these previous studies; we just deployed their approach and experiments on our used dataset. We deployed study [19] which used RF for the thyroid disease prediction. Similarly, we deployed study, [21] which used DT for thyroid disease prediction. Another study [20] is used, which worked on thyroid disease and proposed DNN. Similarly, we deployed the approach proposed in [29] as they worked for the heart disease dataset using a similar type of dataset. The proposed CNN to extract the feature and proposed a hybrid model using three machine learning models stochastic gradient descent classifier, LR, and SVM. We deployed that approach on our dataset also. In comparison with all other studies, our approach performs significantly better as it achieves 0.99 scores in terms of all evaluation parameters. Table 18 shows the comparison between our approach and other studies.

### 4.10. Discussion on Hyperthyroidism and Hypothyroidism

The thyroid disease prediction has been challenging, as the prior detection and evaluation of thyroid symptoms without doctor involvement are not easy. Therefore, thyroid disease classification solutions can accurately predict the thyroid disease type like hyperthyroidism or hypothyroidism, given the machine learning models are trained with sufficient data samples and their performance is optimized. Our work focused on accurately classifying the patient’s thyroid condition given the data samples. Our technique can be incorporated into a software-based solution to enter the patient data, and the software leverages the trained machine learning model to estimate the patient thyroid condition. We are also exploring the additional datasets, which can provide the data samples for other thyroid-related classes like primary, secondary hypothyroid, T3 toxic, secondary toxic, patients’ anti-thyroid treatment status, therapy condition, etc. Our detection method can classify the patient’s disease condition using the proposed machine learning method. In addition, with data from more classes and additional data for existing classes, the performance of the models can be generalized to other thyroid diseases. The proposed approach shows robust results, which can be significantly important for real-time disease detection.

## 5. Conclusions

With an alarming increase in recent years, thyroid disease detection has emerged as an important medical problem and requires efficient automatic prediction models. Existing studies predominantly focus on model optimization and feature engineering and feature selection is less explored. Moreover, the dataset used for model evaluation is small sized and models are not validated. This study overcomes these limitations and proposes an approach that uses feature selection along with machine learning and deep learning models. Besides FFS, BFS, BiDFE, and extra tree classifier-based features, machine learning and deep learning models are employed. Results indicate that extra tree classifier-based selected features tend to provide the highest accuracy of 0.99 when used with the RF model. Other feature techniques yield poor results due to feature reduction, which degrades the performance of both the deep learning and machine learning models, especially linear models. The lower computational complexity of the machine learning models like RF makes them good candidates for thyroid disease prediction. Similarly, 10-fold cross-validation results corroborate these findings. Performance comparison with state-of-the-art approaches indicates the superior performance of the proposed approach. We see the feature reduction and 5-class classification problem as the limitation of the study and intend to increase the number of classes in our future work.

## Figures and Tables

**Table 1:** Summary of the systematic analysis of the state-of-the-art thyroid disease studies.

Authors	Year	Sample Size	Dataset Source	Model	Classes	Evaluation Metrics	Results
[9]	2020	-	ToxCast	LR RF SVM XGB ANN	2	F1-score	(TPO) XGB-83% and (TR) RF-81%
[11]	2018	7200 samples, 21 attributes	UCI	SVM, Multiple Linear Regression(MLR), NB and DT	2	Accuracy	MLR 91.59% SVM 96.04% Naive Bayes 6.31% Decision Trees 99.23%
[12]	2020	7547, 30 features	UCI	multi-kernel SVM	3	Accuracy, Sensitivity, and Specificity	Accuracy (97.49%), Sensitivity (99.05%), and Specificity (94.5%)
[13]	2021	3771 samples, 30 attributes	UCI	DT, KNN, RF, and SVM	4	Accuracy	KNN 98.3% SVM 96.1% DT 99.5% RF 99.81%
[14]	2021	519 samples	diagnostic center Dhaka, Bangladesh	SVM, DT, RF, LR, and NB. Recursive Feature Selection (RFE), Univariate Feature Selection (UFS) and PCA	4	Accuracy	RFE, SVM, DT, RF, LR accuracy—99.35%
[15]	2021	1250 with 17 attributes	external hospitals and laboratories	SVM, RF, DT, NB, LR, KNN, MLP, linear discriminant analysis (LDA) and DT	3	Accuracy	DT 90.13, SVM 92.53 RF 91.2 NB 90.67 LR 91.73 LDA 83.2 KNN 91.47 MLP 96.4
[16]	2021	7200 patients, with 21 features	UCI	multiple MLP	3	Accuracy	multiple MLP 99%
[17]	2021	690 samples, 13 features	datasets from KEEL repo and District Headquarters teaching hospital, Pakistan	KNN without feature selection, KNN using L1-based feature selection, and KNN using chi-square-based feature selection	3	Accuracy	KNN 98%
[18]	2021	3772 and 30 attributes	UCI	RF, sequential minimal optimization (SMO), DT, and K-star classifier	2	Accuracy	K = 6, RF 99.44%, DT 98.97%, K-star 94.67%, and SMO 93.67%
[19]	2022	3163	UCI	DT, RF, KNN, and ANN	2	Accuracy	Best performance Accuracy RF 94.8%
[21]	2022	215 with 5 features	UCI	KNN, XGB, LR, DT	3	Accuracy	KNN 81.25 XGBoost 87.5 LR 96.875 DT 98.59
[20]	2022	3152, 23 features	UCI	DNN	2	Accuracy	Accuracy 99.95%

**Table 2:** Dataset description.

Features	Sample Count
31	9172

**Table 3:** Data sample attribute Types.

Attribute	Description	Data Type
age	age of the patient	(int)
sex	sex patient identifies	(str)
on_thyroxine	whether patient is on thyroxine	(bool)
query on thyroxine	whether patient is on thyroxine	(bool)
on antithyroid meds	whether the patient is on antithyroid meds	(bool)
sick	whether patient is sick	(bool)
pregnant	whether patient is pregnant	(bool)
thyroid_surgery	whether patient has undergone thyroid surgery	(bool)
I131_treatment	whether patient is undergoing I131 treatment	(bool)
query_hypothyroid	whether the patient believes they have hypothyroid	(bool)
query_hyperthyroid	whether the patient believes they have hyperthyroid	(bool)
lithium	whether patient * lithium	(bool)
goitre	whether patient has goitre	(bool)
tumor	whether patient has tumor	(bool)
hypopituitary	whether patient * hyperpituitary gland	(float)
psych	whether patient * psych	(bool)
TSH_measured	whether TSH was measured in the blood	(bool)
TSH	TSH level in blood from lab work	(float)
T3_measured	whether T3 was measured in the blood	(bool)
T3	T3 level in blood from lab work	(float)
TT4_measured	whether TT4 was measured in the blood	(bool)
TT4	TT4 level in blood from lab work	(float)
T4U_measured	whether T4U was measured in the blood	(bool)
T4U	T4U level in blood from lab work	(float)
FTI_measured	whether FTI was measured in the blood	(bool)
FTI	FTI level in blood from lab work	(float)
TBG_measured	whether TBG was measured in the blood	(bool)
TBG	TBG level in blood from lab work	(float)
referral_source		(str)
target	hyperthyroidism medical diagnosis	(str)
patient_id	unique id of the patient	(str)

**Table 4:** Description of the class-wise target.

Condition	Diagnosis Class	Count
hyperthyroid	hyperthyroid (A)	147
	T3 toxic (B)	21
	toxic goiter (C)	6
	secondary toxic (D)	8
hypothyroid	hypothyroid (E)	1
	primary hypothyroid (F)	233
	compensated hypothyroid (G)	359
	secondary hypothyroid (H)	8
binding protein:	increased binding protein (I)	346
	decreased binding protein (J)	30
general health	concurrent non-thyroidal illness (K)	436
replacement therapy:	underreplaced (M)	111
	consistent with replacement therapy (L)	115
	overreplaced (N)	110
antithyroid treatment:	antithyroid drugs (O)	14
	I131 treatment (P)	5
	surgery (Q)	14
miscellaneous:	discordant assay results (R)	196
	elevated TBG (S)	85
	elevated thyroid hormones (T)	0
no condition	(-)	6771

**Table 5:** Balanced dataset for Thyroid disease classification.

Class	Prepossessed Count	Final Count
Normal	6771	400
primary hypothyroid	233	233
increased binding protein	346	346
compensated hypothyroid	359	359
concurrent non-thyroidal illness	436	436

**Table 6:** Sample of dataset.

age	sex	on_thyroxine	query_on_thyroxine	on_antithyroid_meds	sick	pregnant	thyroid_surgery
29	F	f	f	f	f	f	f
71	F	t	f	f	f	f	f
61	M	f	f	f	t	f	f
88	F	f	f	f	f	f	f
**I131_treatment**	**query_hypothyroid**	**query_hyperthyroid**	**lithium**	**goitre**	**tumor**	**hypopituitary**	**psych**
f	t	f	f	f	f	f	f
f	f	f	f	f	f	f	f
f	f	f	f	f	f	f	f
f	f	f	f	f	f	f	f
**TSH_measured**	**TSH**	**T3_measured**	**T3**	**TT4_measured**	**TT4**	**T4U_measured**	**T4U**
t	0.3	f		f		f	
t	0.05	f		t	126	t	1.38
t	9.799999	t	1.2	t	114	t	0.84
t	0.2	t	0.4	t	98	t	0.73
**FTI_measured**	**FTI**	**TBG_measured**	**TBG**	**referral_source**	**target**	**patient_id**	
f		f		other	-	$8.41\times 10^{8}$	
t	91	f		other	I	$8.41\times 10^{8}$	
t	136	f		other	G	$8.41\times 10^{8}$	
t	134	f		other	K	$8.41\times 10^{8}$	

**Table 7:** Target class count for training and testing sets.

Class	Hyper-Parameters	Tuning Range
LR	solver = liblinear, C = 5.0	solver = {liblinear, saga, sag}, C = {1.0 to 8.0}
SVM	kernel = ‘linear’, C = 5.0	kernel = {‘linear’, ‘poly’, ‘sigmoid’} C = {1.0 to 8.0}
RF	n_estimators = 200, max_depth = 20	n_estimators = {10 to 300}, max_depth = {2 to 50}
GBM	n_estimators = 200, max_depth = 20, learning_rat = 0.5	n_estimators = {10 to 300}, max_depth = {2 to 50}, learning_rat = {0.1 to 0.9}
ADA	n_estimators = 200, max_depth = 20, learning_rat = 0.5	n_estimators = {10 to 300}, max_depth = {2 to 50}, learning_rat = {0.1 to 0.9}

**Table 8:** Number of samples for training and test subset.

Target Class	Training	Testing	Total
“_” (0)	325	75	400
F (1)	190	43	233
G (2)	280	79	359
I (3)	271	75	346
K (4)	353	83	436

**Table 9:** Results of machine learning models using original feature set.

Model	Accuracy	Precision	Recall	F1 Score
RF	0.98	0.98	0.98	0.98
GBM	0.97	0.98	0.98	0.98
ADA	0.97	0.97	0.97	0.97
LR	0.85	0.85	0.85	0.85
SVM	0.85	0.85	0.85	0.85

**Table 10:** Performance of machine learning models using FFS feature set.

Model	Accuracy	Precision	Recall	F1 Score
RF	0.97	0.97	0.96	0.96
GBM	0.97	0.97	0.96	0.96
ADA	0.93	0.92	0.92	0.92
LR	0.83	0.83	0.82	0.82
SVM	0.92	0.92	0.92	0.92

**Table 11:** Results using BFE feature set with machine learning models.

Model	Accuracy	Precision	Recall	F1 Score
RF	0.96	0.96	0.95	0.95
GBM	0.92	0.92	0.91	0.91
ADA	0.83	0.84	0.83	0.83
LR	0.83	0.83	0.82	0.82
SVM	0.92	0.92	0.92	0.92

**Table 12:** Performance of models using BiDFE feature set.

Model	Accuracy	Precision	Recall	F1 Score
RF	0.98	0.98	0.98	0.98
GBM	0.96	0.96	0.96	0.96
ADA	0.84	0.87	0.85	0.84
LR	0.81	0.83	0.81	0.81
SVM	0.92	0.92	0.92	0.92

**Table 13:** Performance of models using MLFS feature set.

Model	Accuracy	Precision	Recall	F1 Score
RF	0.99	0.99	0.99	0.99
GBM	0.98	0.98	0.98	0.98
ADA	0.97	0.97	0.97	0.97
LR	0.87	0.88	0.87	0.87
SVM	0.92	0.92	0.92	0.92

**Table 14:** Results of 10-fold cross-validation.

Feature	Model	Accuracy	SD	Time
Original	RF	0.94	+/−0.10	1.689
	GBM	0.93	+/−0.13	3.831
	ADA	0.93	+/−0.08	1.758
	LR	0.84	+/−0.13	0.330
	SVM	0.88	+/−0.12	243.126
FS	RF	0.93	+/−0.10	0.440
	GBM	0.90	+/−0.14	1.349
	ADA	0.89	+/−0.08	0.743
	LR	0.78	+/−0.13	0.330
	SVM	0.90	+/−0.15	210.65
BE	RF	0.93	+/−0.11	0.601
	GBM	0.90	+/−0.14	1.380
	ADA	0.87	+/−0.07	0.635
	LR	0.78	+/−0.13	0.111
	SVM	0.90	+/−0.15	173.80
BiDFE	RF	0.93	+/−0.03	0.677
	GBM	0.90	+/−0.02	8.733
	ADA	0.89	+/−0.06	0.617
	LR	0.78	+/−0.06	0.111
	SVM	0.90	+/−0.04	42.496
ML FS	RF	0.94	+/−0.01	1.689
	GBM	0.93	+/−0.13	3.831
	ADA	0.93	+/−0.08	1.758
	LR	0.84	+/−0.13	0.330
	SVM	0.91	+/−0.13	365.51

**Table 15:** Architecture of deep learning models.

Model	Hyperparameters
LSTM	Embedding (4000, 100, input_length = …) Dropout (0.5) LSTM (128) Dense (5, activation = ‘softmax’)
CNN	Embedding (4000, 100, input_length = …) Conv1D (128, 5, activation = ‘relu’) MaxPooling1D (pool_size = 5) Activation (‘relu’) Dropout (rate = 0.5) Flatten() Dense (5, activation = ‘softmax’)
CNN-LSTM	Embedding (4000, 100, input_length = …) Conv1D (128, 5, activation = ‘relu’) MaxPooling1D (pool_size = 5) LSTM (100) Dense (5, activation = ‘softmax’)
loss = ‘categorical_crossentropy’, optimizer = ‘adam’, epochs = 100, batch_size = 16	

**Table 16:** Deep learning models results with each feature selection technique.

Feature	Model	Accuracy	Precision	Recall	F1 Score
Original	LSTM	0.84	0.84	0.83	0.83
	CNN	0.93	0.94	0.92	0.93
	CNN-LSTM	0.90	0.90	0.88	0.88
FS	LSTM	0.62	0.63	0.59	0.59
	CNN	0.86	0.87	0.84	0.85
	CNN-LSTM	0.77	0.78	0.73	0.74
BE	LSTM	0.57	0.61	0.54	0.54
	CNN	0.86	0.87	0.84	0.84
	CNN-LSTM	0.86	0.87	0.84	0.85
BiDFE	LSTM	0.83	0.83	0.80	0.80
	CNN	0.85	0.84	0.81	0.82
	CNN-LSTM	0.87	0.88	0.84	0.86
ML FS	LSTM	0.57	0.63	0.54	0.55
	CNN	0.89	0.89	0.87	0.88
	CNN-LSTM	0.92	0.91	0.91	0.91

**Table 17:** Deep learning models computational time.

Model	FFS	BFE	BiDFE	MLFS	Original
LSTM	44.975	87.842	98.067	66.361	170.28
CNN	83.088	37.796	131.48	30.852	56.436
CNN-LSTM	150.53	65.992	214.96	47.922	97.662

**Table 18:** Comparison with other studies.

Ref.	Year	Model	Accuracy	F1 Score
[19]	2022	RF	0.98	0.98
[21]	2022	DT	0.98	0.97
[20]	2022	DNN	0.93	0.93
[29]	2022	ConvSGLV	0.96	0.96
This study	2022	MLFS+RF	0.99	0.99

## Data Availability

The dataset is available at https://archive.ics.uci.edu/ml/datasets/thyroid+disease.
